# Author Correction: Polyploidy, regular patterning of genome copies, and unusual control of DNA partitioning in the Lyme disease spirochete

**DOI:** 10.1038/s41467-023-42035-6

**Published:** 2023-10-09

**Authors:** Constantin N. Takacs, Jenny Wachter, Yingjie Xiang, Zhongqing Ren, Xheni Karaboja, Molly Scott, Matthew R. Stoner, Irnov Irnov, Nicholas Jannetty, Patricia A. Rosa, Xindan Wang, Christine Jacobs-Wagner

**Affiliations:** 1 Department of Biology, Stanford University, Palo Alto, CA USA; 2 Sarafan ChEM-H Institute, Stanford University, Palo Alto, CA USA; 3 The Howard Hughes Medical Institute, Palo Alto, CA USA; 4 Laboratory of Bacteriology, Rocky Mountain Laboratories, Division of Intramural Research, National Institute of Allergy and Infectious Diseases, National Institutes of Health, Hamilton, MT USA; 5 Department of Mechanical Engineering, Yale University, New Haven, CT USA; 6 Microbial Sciences Institute, Yale West Campus, West Haven, CT USA; 7 Department of Biology, Indiana University, Bloomington, IN USA; 8 Department of Molecular, Cellular, and Developmental Biology, Yale University, New Haven, CT USA; 9 Present Address: Bacterial Vaccine Development Group, Vaccine and Infectious Disease Organization, University of Saskatchewan, Saskatoon, SK Canada

**Keywords:** Cellular microbiology, Chromosomes, Pathogens, Cellular microbiology

Correction to: *Nature Communications* 10.1038/s41467-022-34876-4, published online 22 November 2022

The original version of this Article contained multiple errors in figures and data files, due to incorrect data transfer from image analysis output files:

1. The original version of this Article contained errors in Fig. 1b. The correct version of Fig. 1 is:
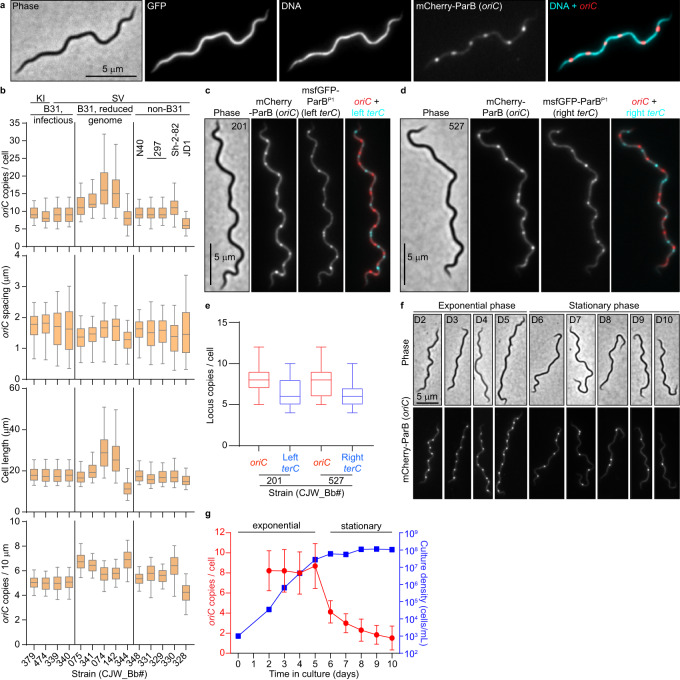


which replaces the previous incorrect version:
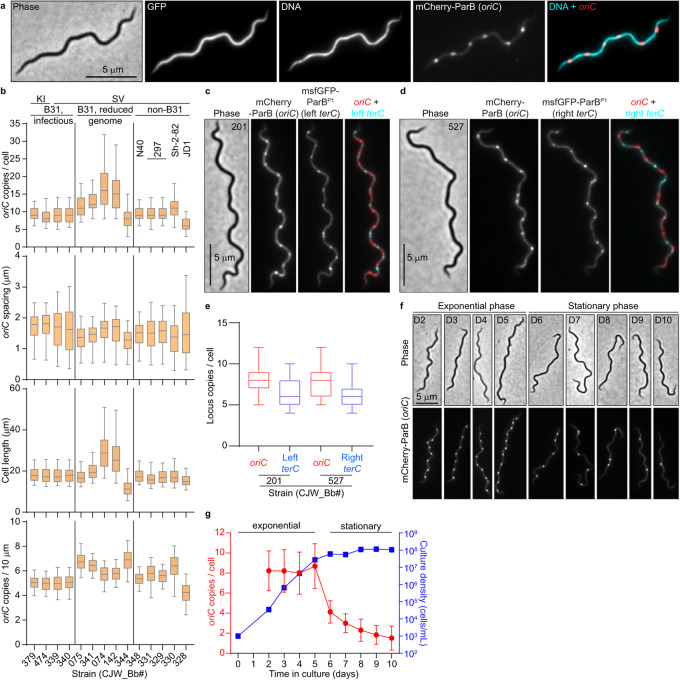


2. The original version of this Article contained errors in Fig. 2b. The correct version of Fig. 2 is:
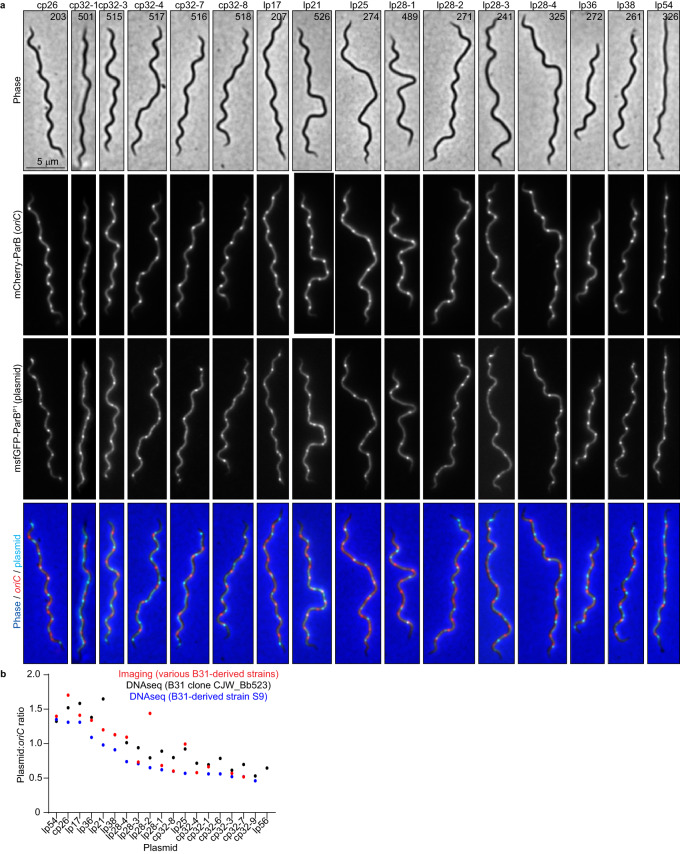


which replaces the previous incorrect version:
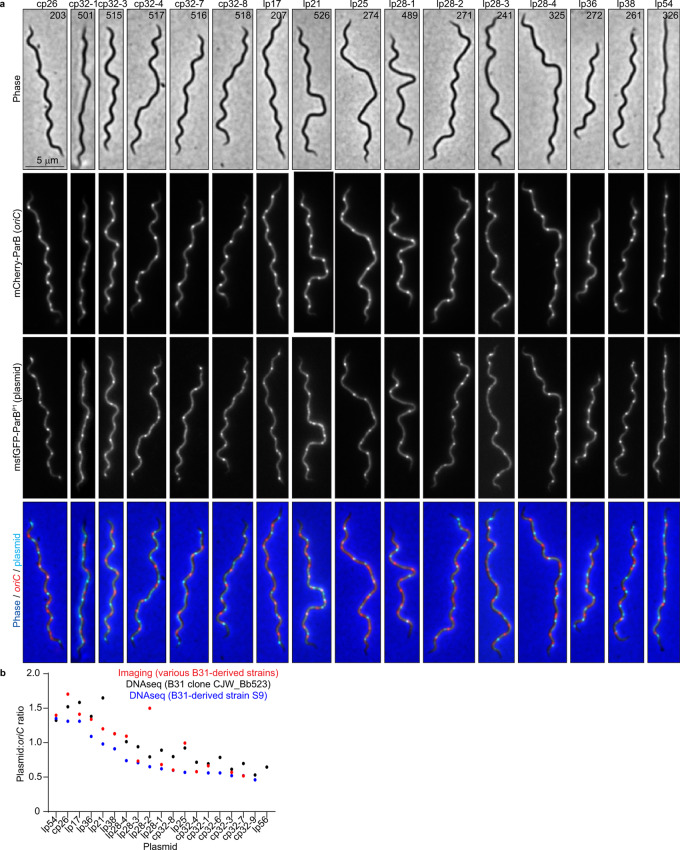


3. The original version of this Article contained errors in Fig. 4d. The correct version of Fig. 4 is:
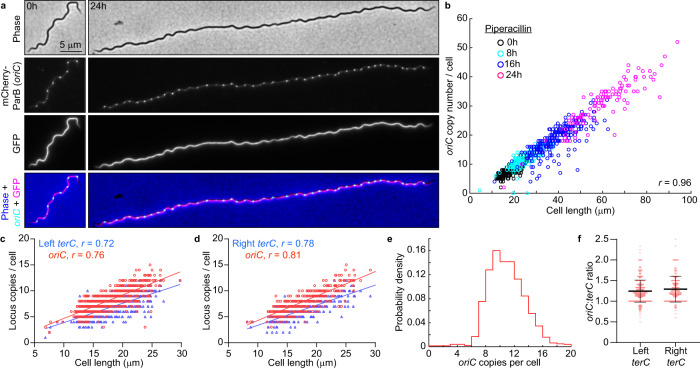


which replaces the previous incorrect version:
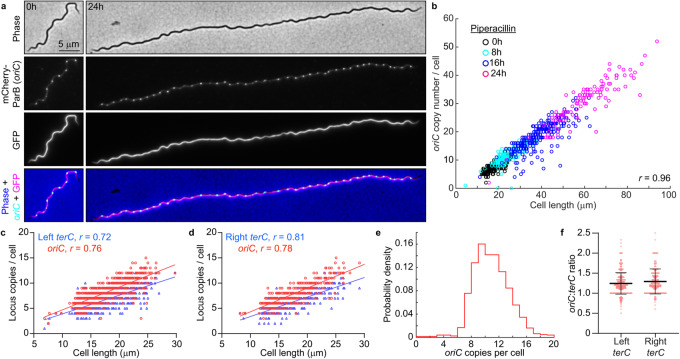


The errors in Figs. 1, 2 and 4 have been corrected in both the PDF and HTML versions of the Article.

4. The original version of the Supplementary Information associated with this Article contained errors in Supplementary Figs. 2a, 3a, c, d and 6e. The HTML has been updated to include a corrected version of the Supplementary Information; the original incorrect versions of these Supplementary Figures can be found as Supplementary Information associated with this Correction.

5. The original version of the Supplementary Information associated with this Article included an incorrect Supplementary Data 2 file and an incorrect Source Data file. The HTML has been updated to include corrected versions of both files; the original incorrect versions of Supplementary Data 2 and Source Data can be found as Supplementary Information associated with this Correction.

### Supplementary information

Original Supplementary Information

Updated Supplementary Information

Original Supplementary Data 2

Updated Supplementary Data 2

Original Source Data

Updated Source Data

