# Supplementary material for: Polyploidy, regular patterning of genome copies, and unusual control of DNA partitioning in the Lyme disease spirochete
Source: Nat Commun. 2022 Nov 22;13:7173. doi: 10.1038/s41467-022-34876-4 (PMC9712426; doi:10.1038/s41467-022-34876-4)
Supplement: Supplementary file 1 — Supplementary Information [file 41467_2022_34876_MOESM1_ESM.pdf]

**Polyploidy, regular patterning of genome copies, and unusual control of DNA partitioning in the Lyme disease spirochete**

Constantin N. Takacs, Jenny Wachter, Yingjie Xiang, Zhongqing Ren, Xheni Karaboja, Molly Scott, Matthew R. Stoner, Irnov Irnov, Nicholas Jannetty, Patricia A. Rosa, Xindan Wang, and Christine Jacobs-Wagner

## SUPPLEMENTARY INFORMATION

**Table of contents……………………………………………………………………………..Page**

Supplementary Notes……………………………………………………………………………...2

Specific labeling of *B. burgdorferi* DNA loci using endogenous and heterologous ParB/*parS* systems…………………...……………………………………………………2

Detection of *oriC* loci in multiple *B. burgdorferi* strains……………………………….....3

Recapitulation of the mouse-tick transmission cycle using strain CJW_Bb474…………4

Supplementary Figure Legends….……………………………………………………………..6

Supplementary Figures…………………………………………………………………………..16

Supplementary References…...…………………………………………………………………..24

## SUPPLEMENTARY NOTES

*Specific labeling of* B. burgdorferi *DNA loci using endogenous and heterologous ParB/*parS *systems*

ParB proteins specifically recognize their cognate *parS* sequence and spread onto adjacent DNA sequences[1-3]. Due to this property, expression of a fluorescent protein-tagged ParB protein leads to accumulation of its fluorescence into a diffraction-limited signal that pinpoints the subcellular location of the DNA locus that contains the *parS* sequence[4].

We have adapted this method for use in *B. burgdorferi*, whose chromosome contains a single predicted *parS* sequence[3] located within the *par* locus, 6 kilobases to the left of *oriC* (Supplementary Fig. 1a). We labeled this *parS* sequence either by replacing the native *parB* gene (*bb0434*) with the *mcherry-parB* translational fusion, yielding knock-in strains (Fig. 1a, Supplementary Fig. 1c, Supplementary Data 1), or by driving expression of *mcherry-parB* or *msfgfp-parB* from a multi-copy shuttle vector (SV) using the weak promoter $P_{0826}$[5] (Supplementary Fig. 1c, Supplementary Data 1). mCherry-ParB fluorescent foci formed only when *parS* was present on the *B. burgdorferi* chromosome (Supplementary Fig. 1b).

To label an additional *B. burgdorferi* locus, we first inserted the *parS* sequence of *E. coli* plasmid P1[2], hereafter referred to as $parS^{P1}$, into the *B. burgdorferi* genome. We then expressed an *msfgfp* fusion to the *parB* gene of plasmid P1 ($msfgfp\text{-}parB^{P1}$) from the same multi-copy shuttle vector that contained the *mcherry-parB* expression cassette (Supplementary Data 1, Supplementary Fig. 1b). We drove expression of $msfgfp\text{-}parB^{P1}$ using the intermediate strength promoter $P_{0031}$[5]. The expressed msfGFP-ParB$^{P1}$ formed fluorescent puncta only when $parS^{P1}$ was also present in a

given *B. burgdorferi* strain (Supplementary Fig. 1b), regardless of whether the chromosomal *parS* site was present or not (Supplementary Fig. 1b,d), confirming that labeling of the two *parS* sequences by their tagged cognate ParB proteins was independent and specific.

This conclusion was further strengthened by quantitative analyses of images of strain CJW_Bb205 (Supplementary Fig. 1d-f). In this strain, mCherry-ParB foci, which pinpoint the subcellular location of *par* loci, and msfGFP-ParB$^{P1}$ foci, which pinpoint the subcellular location of *uvrC* loci (Supplementary Fig. 1a), colocalized almost perfectly (Supplementary Fig. 1d-e). The *par* and *uvrC* loci are 24 kbp away from each other and 6 and 18 kbp away from *oriC*, respectively (Supplementary Fig. 1a). Thus, both labels approximate the subcellular location of *oriC*. Importantly, copy numbers of the *par* and *uvrC* loci were similar (Supplementary Fig. 1f).

*Detection of* oriC *loci in multiple* B. burgdorferi *strains*

We localized the *oriC* locus in several *B. burgdorferi* strains that were derived from the B31 isolate, which is the type strain, as well as from other isolates, namely N40, 297, Sh-2-82, and JD1. For the B31-derived strains, we used the B31-A3-68-Δ*bbe02* genetic background (strains S9 and K2, see Supplementary Data 1), which is easily transformable and fully capable of completing the tick-mouse transmission cycle[6,7]. The S9 derivatives CJW_Bb379 and CJW_Bb474 both carry a replacement of the *parB* gene with an *mcherry-parB* fusion driven by the native *parB* promoter, and are therefore labeled as knock-in (KI) strains (Fig. 1b, Supplementary Fig.1c). CJW_Bb474 additionally expresses free GFP, driven by the $P_{flaB}$ promoter, and inserted into endogenous plasmid cp26 (Fig. 1a, Supplementary Data 1). Strains CJW_Bb339 and CJW_Bb340 are also derived from the infectious K2 and S9 strains,

respectively, but express *mcherry-parB* as a second *parB* copy, in trans, from a shuttle vector (Supplementary Data 1). Strains CJW_Bb339, CJW_Bb340, CJW_Bb379, and CJW_Bb474 each has an almost complete complement of endogenous plasmids. They only lack plasmids cp9, lp5, and lp56 (Supplementary Data 1), which are also absent from the parental strains S9 and K2 and are not required for completion of the tick-mouse transmission cycle[6,8-10]. We therefore refer to these strains as having an infectious background, which we experimentally demonstrated for strain CJW_Bb474 (see below).

We determined that the other B31-derived strains have lost multiple endogenous plasmids (Supplementary Data 1) during their generation and/or the generation of their parental strains[11-13]. At most, strain CJW_Bb075 carries 11 endogenous plasmids, while strain CJW_Bb344 only carries two endogenous plasmids, cp26 and cp32-3 (Supplementary Data 1). They all expressed tagged ParB proteins (mCherry-ParB or msfGFP-ParB) from a shuttle vector (Supplementary Data 1). Lastly, we localized *oriC* loci in several other *B. burgdorferi* strains, including the widely studied N40, 297, and JD1 isolate backgrounds (Fig. 1b, Supplementary Fig. 1c, Supplementary Data 1). We did not determine the endogenous plasmid content of the clones derived from the non-B31 isolates as there are no available characterized sets of primers for multiplex PCR detection of the native plasmids of these strains.

*Recapitulation of the mouse-tick transmission cycle using strain CJW_Bb474*

Strain CJW_Bb474 was used to image the chromosomal copy number in the tick (Fig. 3). Since this strain carries genetic modifications, it was important to assess whether it can reproduce the mouse-tick transmission cycle. Two modifications, inactivation of gene *bbe02* and constitutive

expression of GFP from cp26, did not affect *B. burgdorferi*'s ability to complete its transmission cycle when previously tested in several strain backgrounds[6,7,9,10,14,15]. The third modification, replacement of *parB* with *mcherry-parB*, has not been previously tested. Supplementary Fig. 1i depicts our experimental setup. Mice were infected with *B. burgdorferi* by needle inoculation (step a). Naïve tick larvae were allowed to feed on these infected mice and thus to acquire *B. burgdorferi* (step b). These colonized larvae molted into unfed nymphs (step c), which were then allowed to feed on and transmit *B. burgdorferi* to naïve mice (step d). Infection of mice was confirmed by tissue biopsy culture in BSK-II medium (stages I and V). *B. burgdorferi* acquisition by, and stable colonization of, ticks were assessed in fed larvae, unfed nymphs, and fed nymphs (stages II through IV) by crushing ticks in BSK-II then using the resulting tick extracts to inoculate liquid BSK-II cultures or embedding them in semisolid BSK-agarose plates. Spirochete outgrowth in the BSK-II medium or colony formation in the BSK-agarose plates were deemed evidence that the ticks were colonized by *B. burgdorferi*. All the mice exposed to strain CJW_Bb474, as well as those exposed to the CJW_Bb473 control strain, which only expresses GFP from cp26, were successfully infected (Supplementary Fig. 1j). All the ticks exposed to CJW_Bb474 were also infected, as were most of the ticks exposed to the CJW_Bb473 control (Supplementary Fig. 1j). Additionally, spirochete loads in unfed nymphs were close to $10^2$ cfu/tick for both strains (Supplementary Fig. 1k). These loads increased to above $10^5$ cfu/tick in fed nymphs assayed 10 days after completion of nymphal feeding (Supplementary Fig. 1k). The spirochete burdens that we measured in unfed and fed nymphs are similar to those previously measured in ticks colonized with the parental strain S9[16-18]. These results indicate that strain CJW_Bb474 is fully capable of completing the mouse-tick transmission cycle.

## SUPPLEMENTARY FIGURE LEGENDS

**Supplementary Figure 1. *B. burgdorferi* cells carry multiple chromosome copies**

**a.** Schematic of chromosomal loci localized in this study (not drawn to scale). *oriC* was localized either by labeling the *par* locus through the expression of fluorescently tagged ParB (red) or by insertion of $parS^{P1}$ downstream of *uvrC*, which is located 52% along the length of the chromosome, followed by expression of msfGFP-ParB$^{\mathrm{P1}}$. The telomeres were labeled by insertion of $parS^{P1}$ at the *phoU* or *lptD* loci, which are located at 5% or 98% along the length of the chromosome, respectively, followed by expression of msfGFP-ParB$^{\mathrm{P1}}$. Distances between the labeled DNA loci and the *oriC* or *terC* loci, are shown in kilobase pairs (kbp).

**b.** Images showing that mCherry-ParB and msfGFP-ParB$^{\mathrm{P1}}$ specifically recognize their cognate *parS* sites. mCherry-ParB and msfGFP-ParB$^{\mathrm{P1}}$ were expressed from the same shuttle vector (see methods). The strains are, from top to bottom: CJW_Bb211, CJW_Bb534, CJW_Bb532, and CJW_Bb533. Presence of endogenous *parB* or *parS*, and chromosomal insertion of $parS^{P1}$ are indicated at the left.

**c.** Localization of mCherry-ParB or msfGFP-ParB at *oriC* regions in various strain backgrounds. Tagged ParB was expressed either by knock-in of *mcherry-parB* at the gene locus or in trans, from a shuttle vector. Strain CJW_Bb142 expressed *msfgfp-parB* from a shuttle vector. Strain backgrounds are shown at the left. The CJW_Bb number of each strain is listed on the phase-contrast image.

**d.** Images of a cell of strain CJW_Bb205 showing the *oriC* region co-labeled by expression of mCherry-ParB, which binds to the endogenous *parS* site located within the *par* locus, and msfGFP-ParB$^{\mathrm{P1}}$, which binds to the $parS^{P1}$ sequence introduced at the *oriC*-proximal *uvrC* locus, as shown in (a).

**e.** Fluorescence intensity profiles along the cell length for the cell shown in (d).

**f.** Boxplot showing the number of *oriC* copies per cell based on the labeling of the *par* locus (red) or of the *uvrC* locus (blue) in strain CJW_Bb205. Shown are the mean of the data (middle line), the 25 to 75 percentiles of the data (box), and the 2.5 to 97.5 percentiles of the data (whiskers).

**g.** Images showing DNA fluorescence in situ hybridization (FISH) staining of the repetitive sequence found within the right telomere of the chromosome of strain 297. Strain B31e2, which does not contain this repetitive sequence, serves as a negative control. Cell outlines are in green.

**h.** qPCR-based quantification of chromosomal copy numbers per cell in strain CJW_Bb339 at different culture densities. *flaB* and *recA* gene copy numbers per cell (mean ± standard deviations of measurements done in three replicate cultures) are shown in blue and red, respectively. Culture densities (mean ± standard deviation) are in black. Values do not account for losses that may have occurred during sample prep.

**i.** Schematic of the experimental workflow used to test the transmission of *B. burgdorferi* strains CJW_Bb473 and CJW_Bb474 between ticks and mice. Roman numerals depict the stages at which infection of mice or colonization of ticks by *B. burgdorferi* was assessed.

**j.** Summary of infection or colonization readouts as assayed at the stages depicted in (i). Assay methods are given for each stage. Shown are numbers of positive animals (mice or ticks) over numbers of assayed animals.

**k.** Plot showing *B. burgdorferi* loads in nymphs prior to nymphal stage feeding (unfed) or 10 days after nymphal feeding drop-off (fed). Individual data points, as well as the mean values ± standard deviation, are plotted. For unfed nymphs colonized with strain CJW_Bb473, one of the

nymphs contained no spirochetes (see Source Data). This data point could not be plotted on a log scale but is included in the calculation of the mean.

Source data for panels f, h, and k are provided as a Source Data file. The numbers (*n*) of cells analyzed and the number of replicates are provided in Supplementary Data 2.

**Supplementary Figure 2. *B. burgdorferi* contains multiple copies of its endogenous plasmids**

**a**. Boxplots showing the quantification of various characteristics (plasmid copies per cell; plasmid copies per 10 µm of cell length; plasmid to *oriC* ratios; *oriC* copies per cell; *oriC* copies per 10 µm of cell length, and cell length) for strains in which an endogenous plasmid is labeled by insertion of $parS^{P1}$ and expression of msfGFP-$ParB^{P1}$, while *oriC* is labeled by expression of mCherry-ParB. Strains are, from left to right: CJW_Bb207, CJW_Bb526, CJW_Bb274, CJW_Bb489, CJW_Bb271, CJW_Bb241, CJW_Bb325, CJW_Bb272, CJW_Bb261, CJW_Bb326, CJW_Bb203, CJW_Bb501, CJW_Bb515, CJW_Bb517, CJW_Bb516, and CJW_Bb518. Selected images for each of these strains are provided in Fig. 2a. Shown are the mean of the data (middle line), the 25 to 75 percentiles of the data (box), and the 2.5 to 97.5 percentiles of the data (whiskers).

**b.** An exponentially growing culture of strain CJW_Bb203, in which *oriC* is labeled by expression of mCherry-ParB and cp26 is labeled using the msfGFP-$ParB^{P1}$/$parS^{P1}$ system, was diluted to $10^3$ cells/mL, then imaged daily from day 4 through day 8 of growth in culture. Shown is the *oriC* copy number per cell (red, mean ± standard deviation), the cp26 copy number per cell (blue, mean ± standard deviation) and the culture density (black, in cells/mL) at the indicated times.

Source data are provided as a Source Data file. The numbers (*n*) of cells analyzed and the number of replicates are provided in Supplementary Data 2.

**Supplementary Figure 3. Chromosome and plasmid copy numbers correlate with cell length**

**a**. Correlations between *oriC* copy number per cell and cell length in the indicated strains, which are also shown and analyzed in Fig. 1b and Supplementary Fig. 1c. *r*, Spearman's correlation coefficient.

**b.** Same as in (a), except for strains CJW_Bb074 and CJW_Bb142. These strains have longer characteristic cell lengths, which is reflected in the range used for the x-axis.

**c.** Correlations between plasmid copy number per cell and cell length in a subset of the strains described in Supplementary Fig. 2a and Fig. 2a. The analyzed plasmid is listed in blue, while the Spearman's correlation coefficient *r* is in burgundy.

**d.** Correlations between plasmid copy number per cell and cell length in the remaining strains described in Supplementary Fig. 2a and Fig. 2a and not included in (c). These plasmids have fewer copies per cell. The analyzed plasmid is listed in blue, while the Spearman's correlation coefficient *r* is in burgundy.

Source data are provided as a Source Data file. The numbers (*n*) of cells analyzed and the number of replicates are provided in Supplementary Data 2.

**Supplementary Figure 4. ParZ is a novel centromere-binding protein that controls oriC segregation**

**a.** Whole genome ChIP-seq profiles for strains expressing free GFP (CJW_Bb473), ParZ-msfGFP (CJW_Bb378), mCherry-ParB (CJW_Bb379), or ParA-msfGFP (CJW_Bb488). The x-axis shows the chromosome coordinates followed by the concatenated endogenous plasmids of strain S9 in the order: lp28_3, lp25, lp28_2, lp38, lp36, lp28_4, lp54, cp26, lp17, lp28_1, cp32_1, cp32_3, cp32_4, cp32_6, cp32_7, cp32_8, cp32_9, and lp21. The vertical dotted lines indicate the boundary between chromosomal and plasmid sequences in the concatenated genome. Two replicates are shown for each strain. No ChIP-seq peaks are seen in the free GFP control. The peaks visible in the other traces correspond to the *par* locus (also see Fig. 6). The endogenous $P_{flaB}$ and *flaB*t sequences were computationally removed from the *flaB* locus on the chromosome sequence before read mapping to prevent erroneous mapping of ChIP-seq reads to the *flaB* locus (see the Online Methods for a detailed explanation).

**b.** ChIP-seq profiles of ParZ-msfGFP and mCherry-ParB binding to the *par* locus in strain CJW_Bb403, which expresses both protein fusions.

**c.** ChIP-seq profile of ParZ-msfGFP binding to the genome of strain CJW_Bb101, which carries *parZ-msfgfp* on a shuttle vector. The two peaks of binding are at the chromosomal *par* locus and on the shuttle vector, as indicated. See panels (d) and (e) for detailed views of binding to these genome regions. Strain CJW_Bb101 lacks all endogenous plasmids except lp54, cp26, lp17, cp32-1, cp32-3, and cp32-4. The sequences corresponding to these plasmids were concatenated in this order, followed by the sequence of the pBSV2G_$P_{0826}$-RBS-ParZ-msfGFP$^{Bb}$ shuttle vector to generate the plasmid portion of the genome of strain CJW_Bb101. The vertical dotted line indicates the boundary between chromosomal and plasmid sequences in the concatenated

genome. The endogenous $P_{0826}$ and $P_{flgB}$ sequences were removed from the chromosome before the mapping of the reads to prevent erroneous mapping of ChIP-seq reads to these chromosomal loci (see the Online Methods for a detailed explanation).

**d.** Detailed view of panel (c) showing the binding of ParZ-msfGFP to the chromosomal *par* locus.

**e.** Detailed view of panel (c) showing the binding of ParZ-msfGFP to sequences within the shuttle vector.

**f.** Images of a cell of strain CJW_Bb101. Arrowheads pinpoint four of the many densely packed ParZ-msfGFP puncta that can be detected in cells of this strain.

**g.** Images of a cell of a strain CJW_Bb571 which expresses ParZ-msfGFP from the *parZ* gene locus and carries an empty shuttle vector.

**h.** ChIP-seq profiles showing an overlay of the landscape binding pattern of free GFP, ParZ-msfGFP, mCherry-ParB, and ParA-msfGFP to the concatenated endogenous plasmid sequences. Traces are those of Replicate 2 shown in (a).

**<u>Supplementary Figure 5. Phylogenetic analyses of Par proteins</u>**

**a.** Alignment of the indicated chromosomally expressed ParB sequences. ParB domains are highlighted at the bottom.

**b.** Alignment of indicated chromosomally expressed ParA sequences. The numbers at the right indicate the same species as those listed in (a), at the right.

**c.** Table showing the distance in base pairs (bp) between *parA* and *parB* homologs in the *par* loci of representative spirochete bacteria. *parA* and *parB* are found in the same orientation with a

short genomic distance separating the two genes, which is suggestive of an operon structure. A negative value indicates overlap of the coding regions of the two genes.

**d.** Organization of the *par* loci of the indicated Lyme disease spirochete strains as visualized using the BorreliaBase genome browser[19].

**e.** Alignment of 65 Borreliaceae ParZ sequences. Putative ParZ domains are highlighted at the bottom. Sequences belonging to Lyme disease and relapsing fever spirochetes are marked at the right.

**Supplementary Figure 6. Characterization of *B. burgdorferi* strains expressing tagged Par proteins and/or carrying *par* gene mutations.**

**a.** ParA-msfGFP signal concentrations in individual cells of strains carrying *parA-msfgfp* as the single *parA* copy at its native locus (knock-in strains). *par* locus mutations present in these strains are indicated at the bottom. From left to right, the strains are: CJW_Bb488, CJW_Bb520, CJW_Bb519, CJW_Bb610. Shown are individual data points as well as means ± standard deviations. A.U., arbitrary units.

**b.** Same as in (a), but also including strains carrying *parA-msfgfp* on a shuttle vector. From left to right, the strains are: CJW_Bb488, CJW_Bb520, CJW_Bb519, CJW_Bb610, CJW_Bb219, CJW_Bb218, CJW_Bb256, and CJW_Bb255. Please note the y-axis range is different than in (a).

**c.** Phase contrast and fluorescence micrographs of strains expressing ParA-msfGFP from a shuttle vector and carrying the indicated *par* locus mutations. From top to bottom, the strains are: CJW_Bb219, CJW_Bb218, CJW_Bb256, and CJW_Bb255.

**d.** Plot showing relative mRNA levels for *parZ* and *parZΔN20* determined by qRT-PCR in the indicated strains. Shown are the individual values and the means from two replicates. Mann-Whitney test comparing *parZΔN20* expression (strain CJW_Bb610) with *parZ* expression (strain CJW_Bb488) yielded $p = 0.33$.

**e.** Plots comparing cell length, *oriC* copy numbers per cell, *oriC* copies per 10 µm of cell length, and abundance of cells without *oriC* foci for control and mutant strains analyzed in Figs. 6, 8, and 9. The boxplots depict the mean of the data (middle line), the 25 to 75 percentiles of the data (box), and the 2.5 to 97.5 percentiles of the data (whiskers). The numbers on the bottom graph represent the number of cells without *oriC* foci and the total number of cells analyzed for each strain. The nature of the *oriC* label is listed at the top. Mutations introduced into the strains are listed at the bottom. From the left, the following strains were used: CJW_Bb378, CJW_Bb490, CJW_Bb524, CJW_Bb616, CJW_Bb603, CJW_Bb602, CJW_Bb379, CJW_Bb525, CJW_Bb626, and CJW_Bb604.

**f.** Phase contrast and fluorescent images of a cell of strain CJW_Bb626 that expresses mCherry-ParB and carries the Δ*parZ* mutation.

**g.** Plot showing the growth kinetics of the indicated strains in semisolid BSK-agarose media. Approximately 50 cells of each strain were plated in triplicate. The plates were then inspected daily from day 5 after plating onwards and visible colonies were counted on each day. The plating efficiency measured on a given day was calculated by dividing the number of colonies counted on that day by the maximum number of colonies counted on the same plate during the 9-day course of the experiment. Shown are means ± standard deviations.

**h.** Growth curves of the indicated strains in BSK-II medium. Cultures were inoculated in triplicate from exponentially growing parental cultures and then cell densities were determined daily by direct counting under darkfield illumination. Shown are means ± standard deviations. Source data for panels a,b,d,e,g, and h are provided as a Source Data file. The numbers (*n*) of cells analyzed and the number of replicates are provided in Supplementary Data 2.

**Supplementary Figure 7. ParZ-like sequences can be found in Firmicutes, Fusobacteria, and their phages**

Blast searches were performed using *B. burgdorferi* ParZ as bait. No hits were obtained among archaeal and eukaryotic proteins. Hits obtained among bacterial chromosome-encoded proteins are in red, while those obtained among bacteriophage-encoded proteins are in cyan. Letters highlight the Firmicutes and Fusobacteria phyla, or the Borreliaceae family, while the numbers highlight the indicated genera.

**Supplementary Figure 8. Schematic pedigree of genetic changes engineered at the *par* and *smc* loci**

**a.** Depiction of genetic changes at the *par* locus. Genes affected by genetic modifications are in orange. Genes flanking the modified region and not affected by the changes are in gray. WT, wild type. *aacC1*, gentamicin resistance cassette. *aphI,* kanamycin resistance cassette. The promoters and transcriptional terminators present in the antibiotic resistance cassettes are not shown. Features are not drawn to scale. The lines starting from the WT locus at the left depict the order in which successive genetic modifications were introduced. *, *parZ*Δ*N20*.

**b.** Same as in (a) but for the *smc* locus. *hyp*, gene *bb0044* of hypothetical function. *, a short sequence encoding the C-terminus of SMC was not deleted to avoid inactivating the promoter upstream of gene *bb0044*.

a. and b. Please note that individual strains (see Supplementary Data 1) may carry genetic modification at a single locus or multiple loci, including the chromosomal *phoU*, *uvrC*, or *lptD* loci, or plasmid-specific loci.

**Supplementary Figure 1**

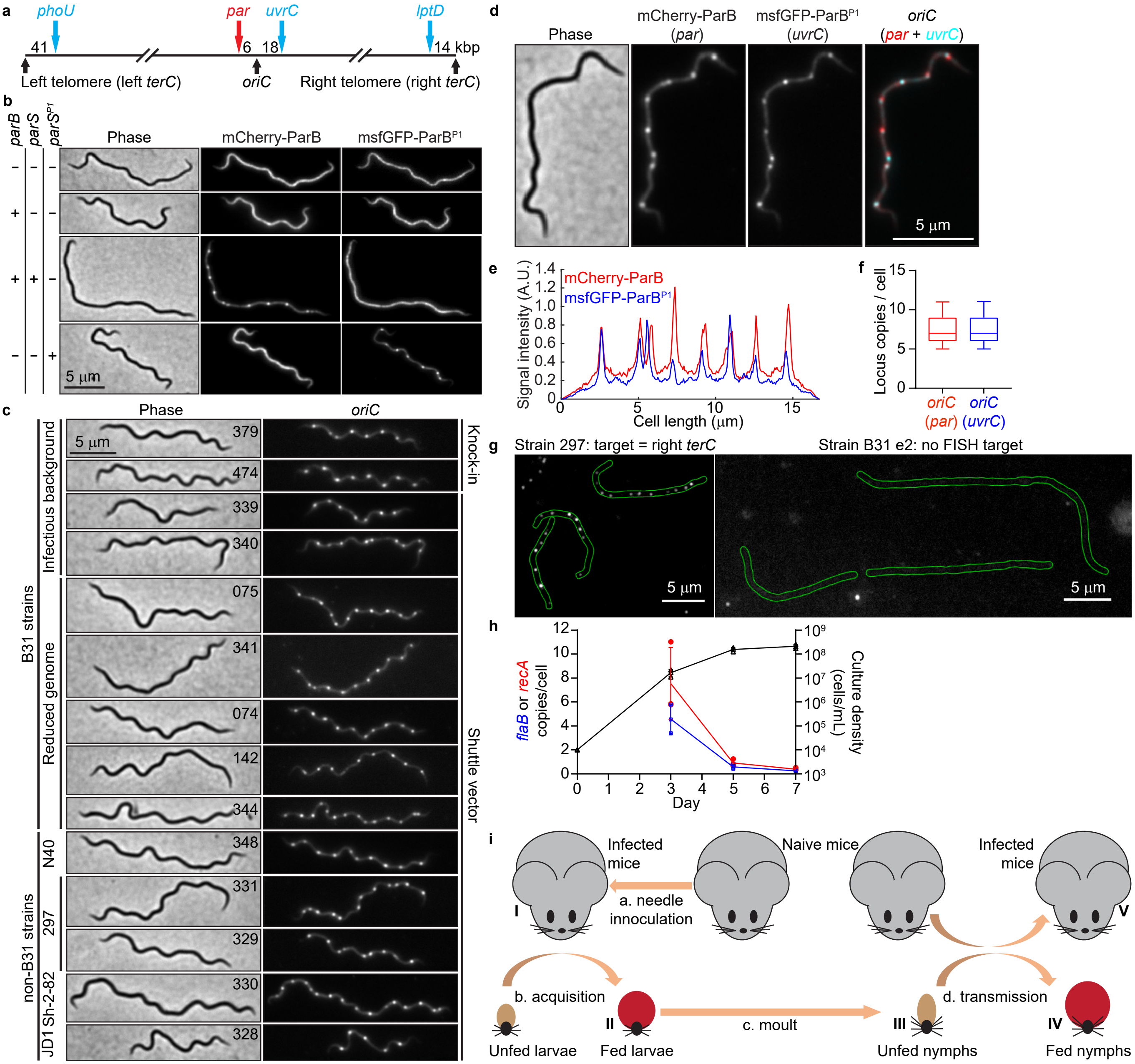


j

| Strain | Genetic Features | Stage (Method) | | | | |
|---|---|---|---|---|---|---|
| | | I (Culture) | II (Culture/Plating) | III (Culture/Plating) | IV (Culture/Plating) | V (Culture) |
| CJW_Bb473 | *gfp* | 5/5 | 5/9 | 5/6 | 6/6 | 2/2 |
| CJW_Bb474 | *gfp ; mcherry-parB* | 5/5 | 9/9 | 6/6 | 6/6 | 2/2 |

k

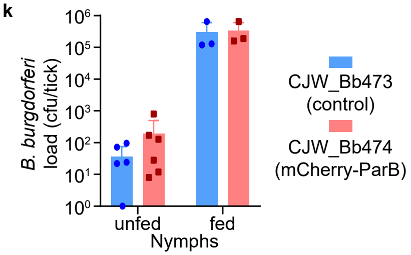

**Supplementary Figure 2**

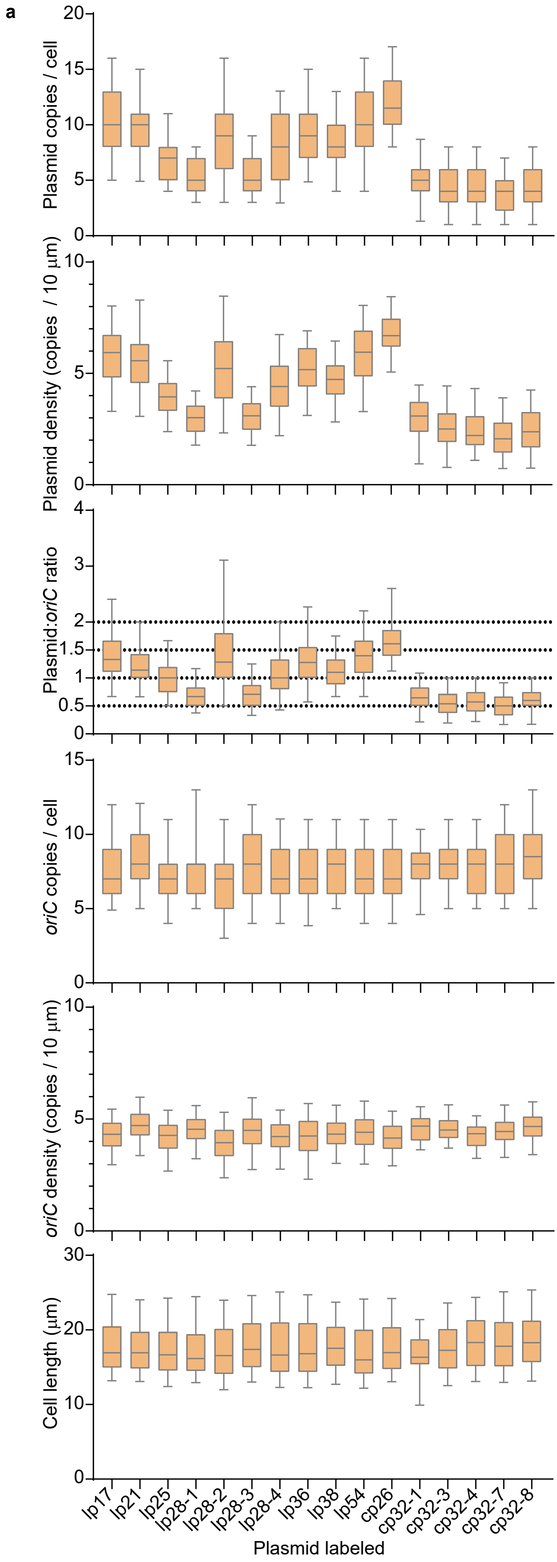


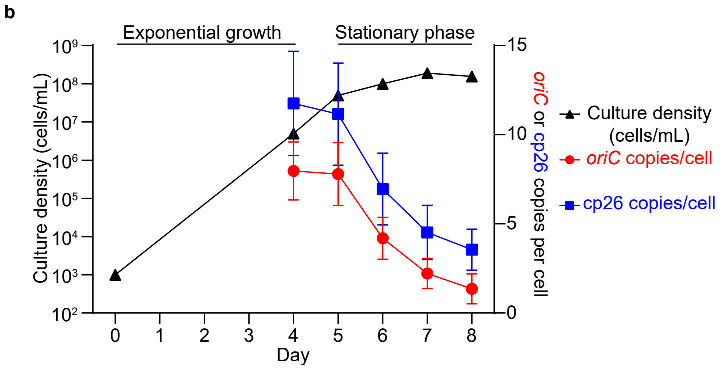

**Supplementary Figure 3**

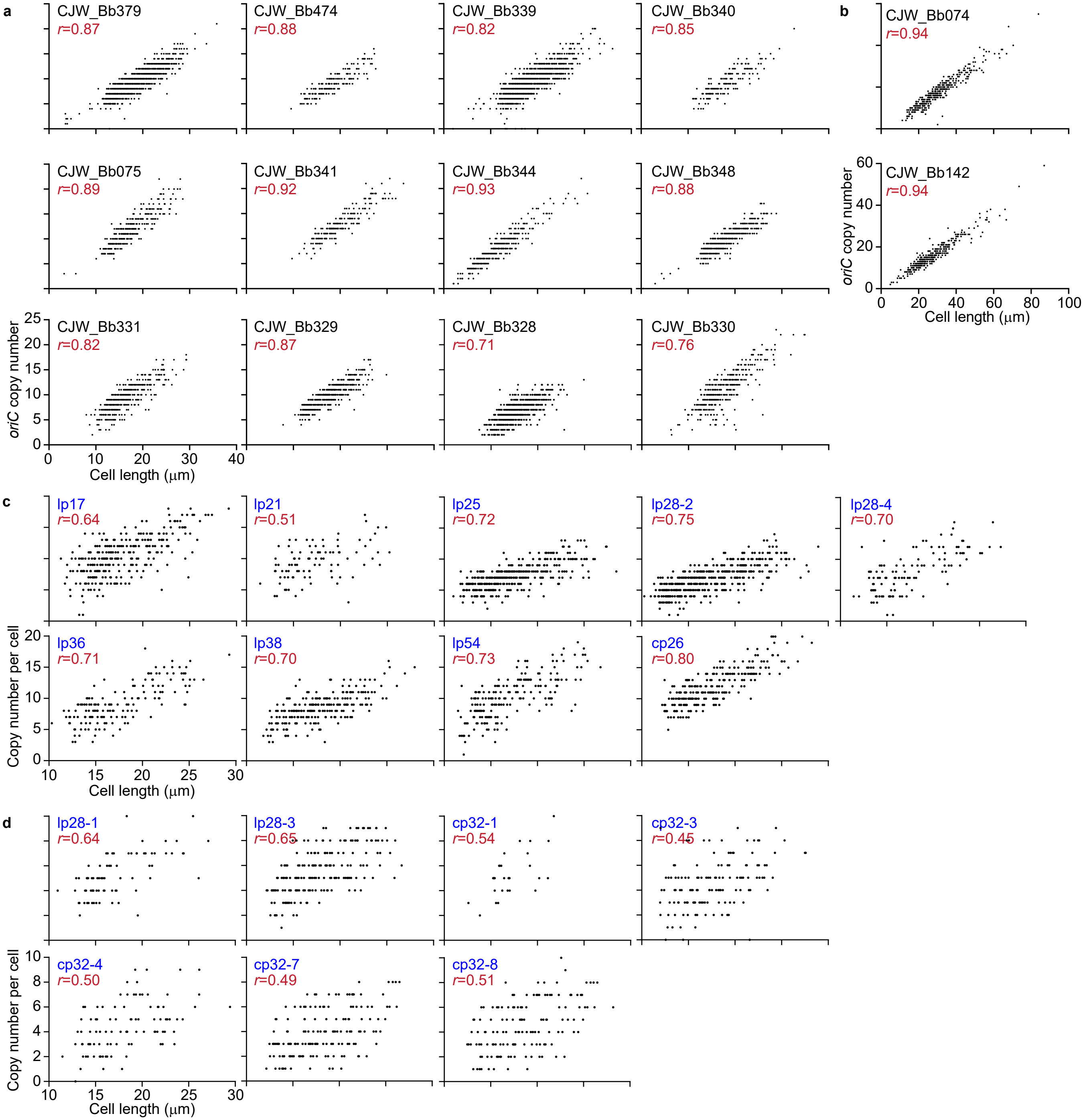

**Supplementary Figure 4**

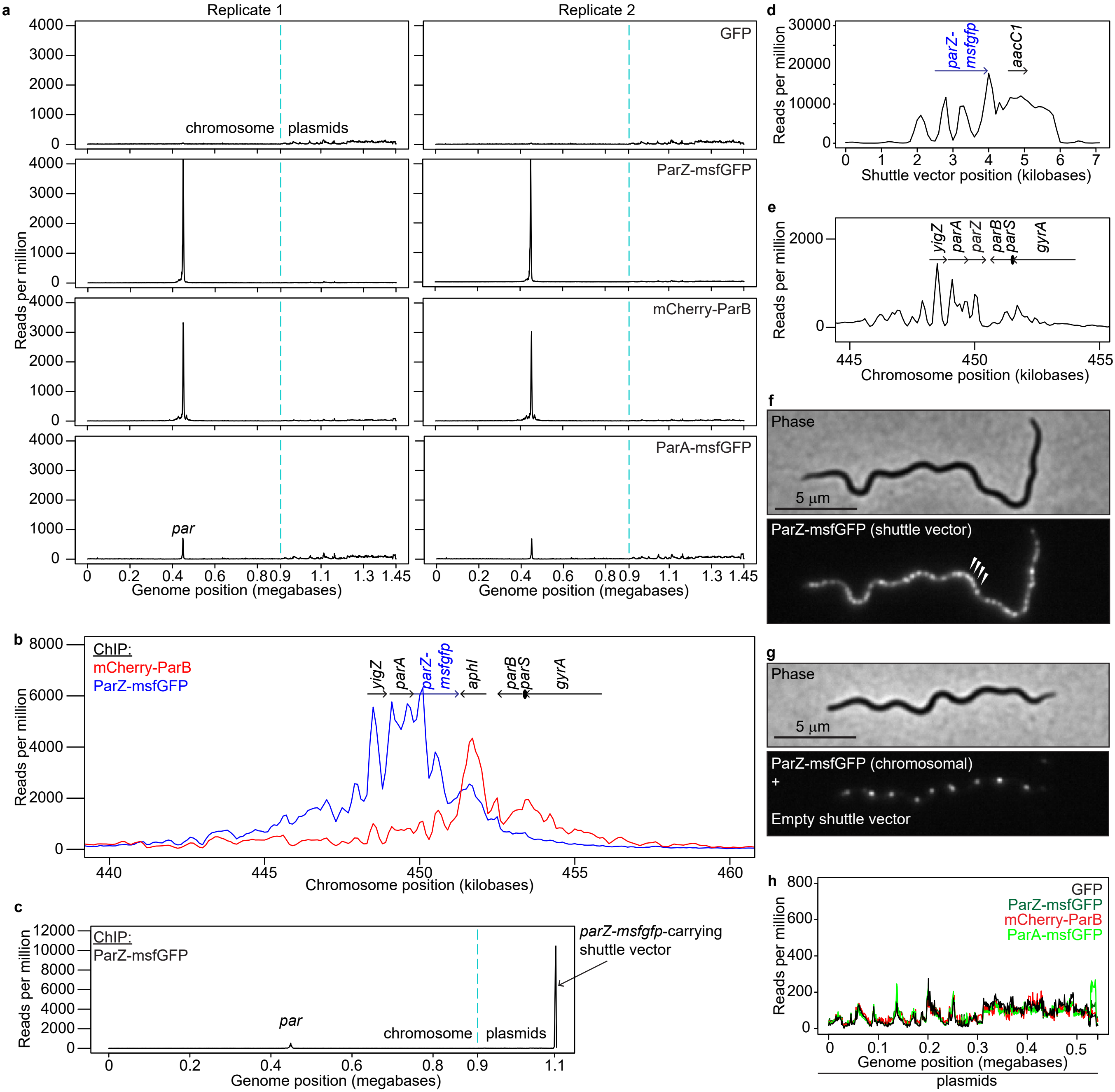

**Supplementary Figure 5**

**a**

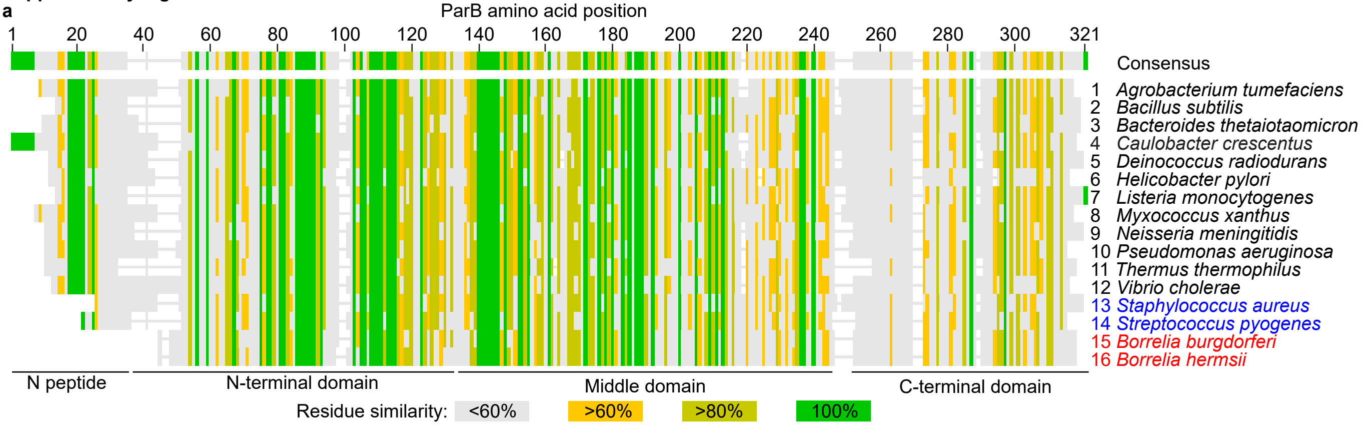


**b**

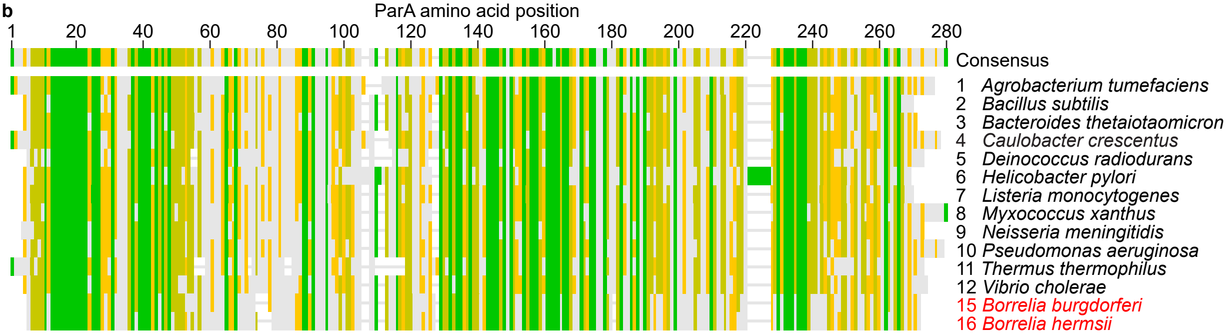


**c**

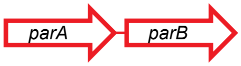


| Species (strain) | *parA-parB* distance (bp) |
|---|---|
| *Brachyspira hyodysenteriae* (WA1) | 100 |
| *Leptospira interrogans* ser. *Copenhegani* (Piyasena) | 3 |
| *Leptospira biflexa* ser. *Patoc* (Patoc 1) | 4 |
| *Treponema denticola* (ATCC 35405) | -7 |
| *Treponema pallidium* subsp. *pallidum* (Nichols) | -13 |
| *Spirochaeta africana* (DSM 8902) | -22 |
| *Sphaerochaeta globosa* (Buddy) | -19 |
| *Salinispira pacifica* (L21-Rpul-D2) | 32 |

**d**

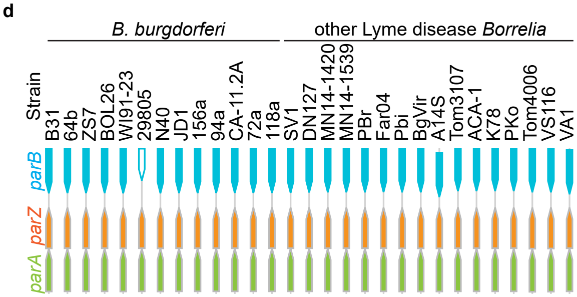


**e**

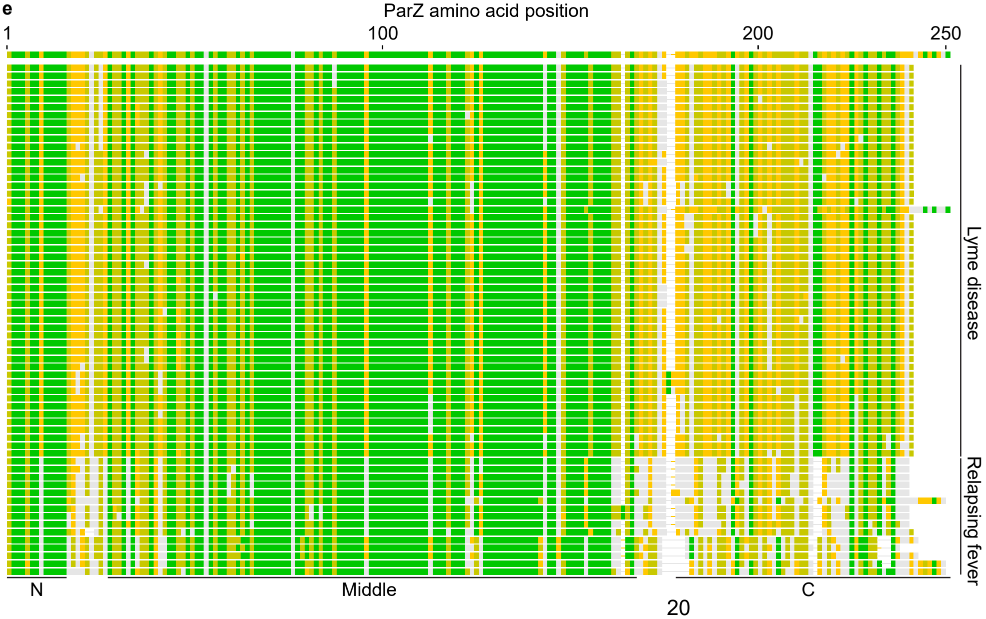

**Supplementary Figure 6**

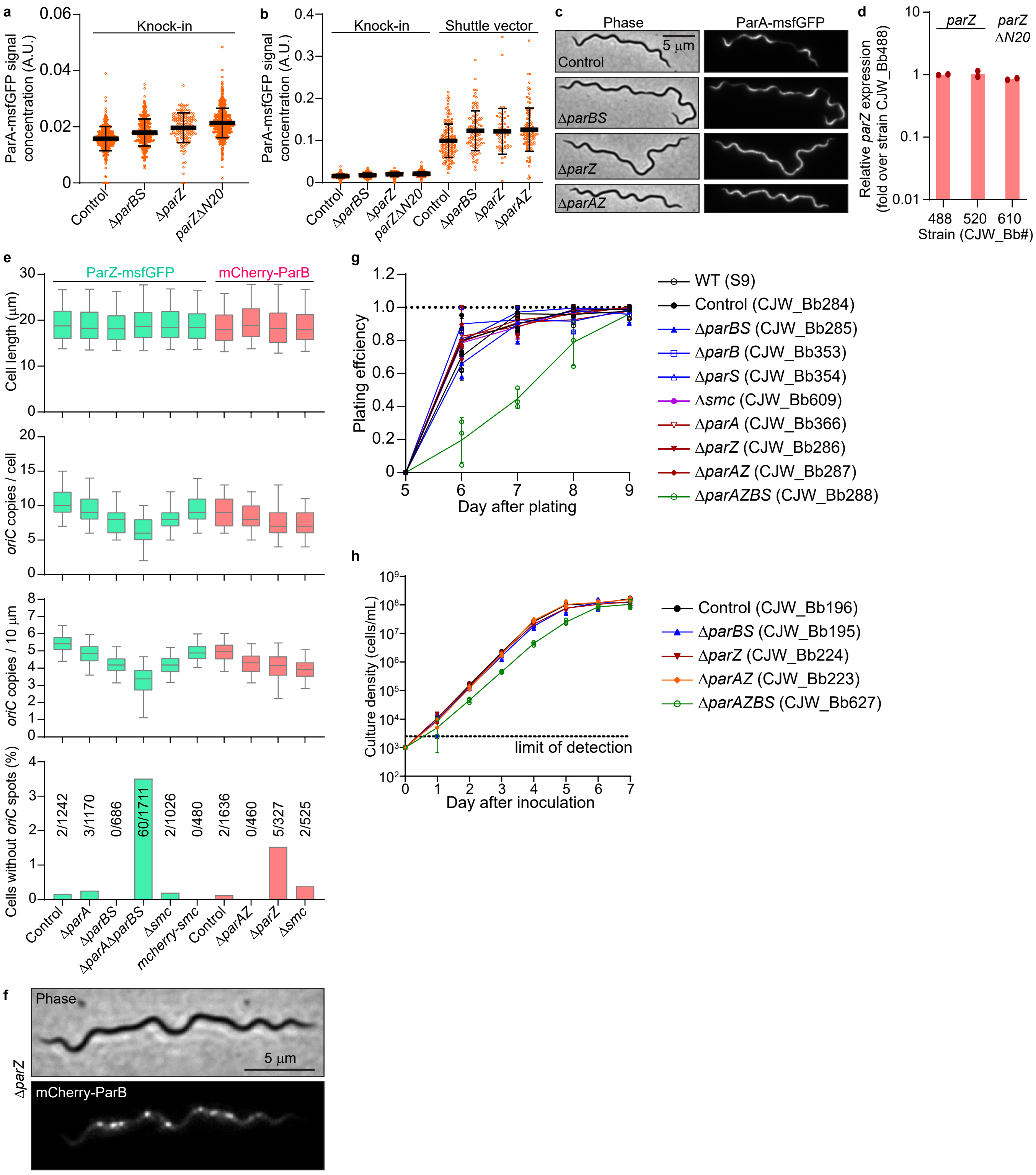

**Supplementary Figure 7**

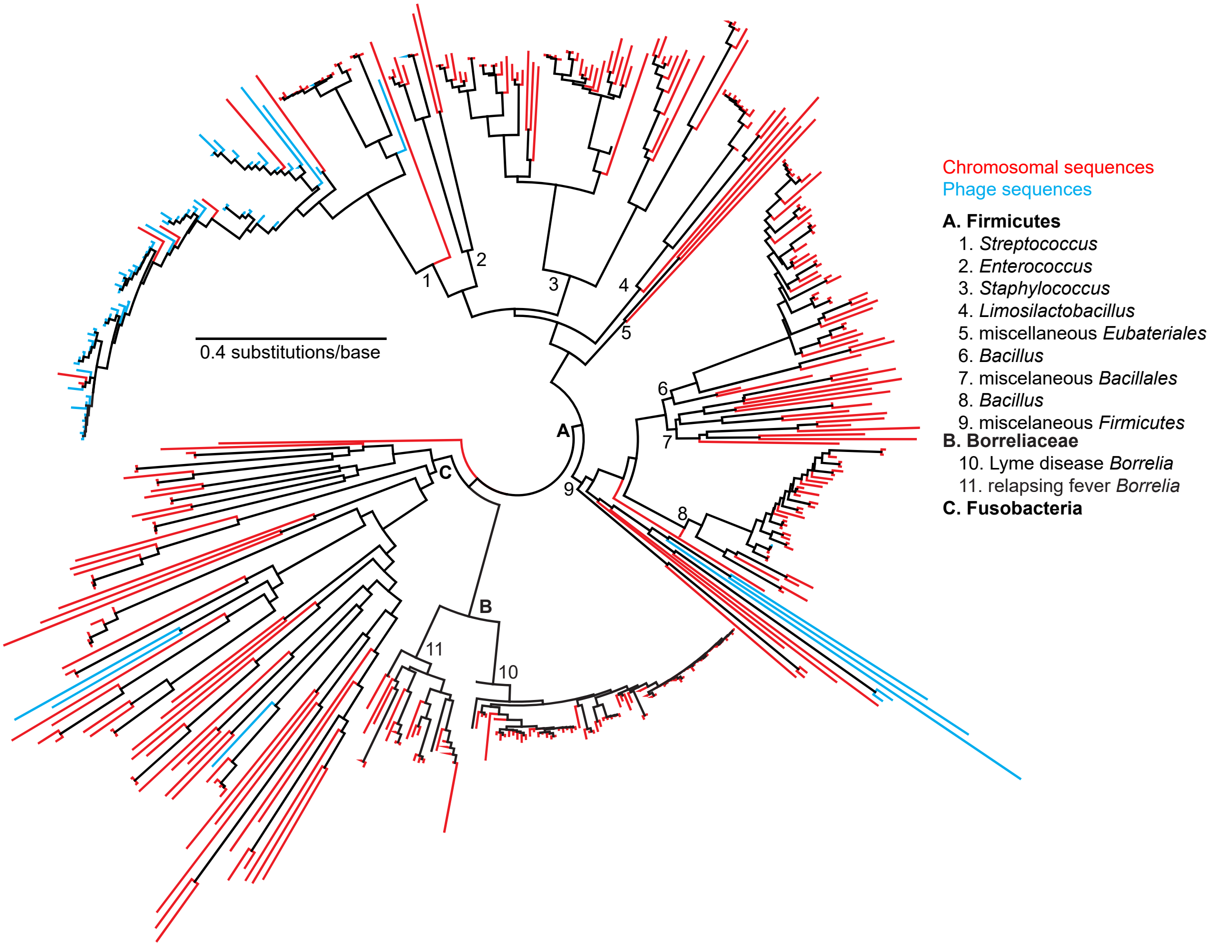

**Supplementary Figure 8**

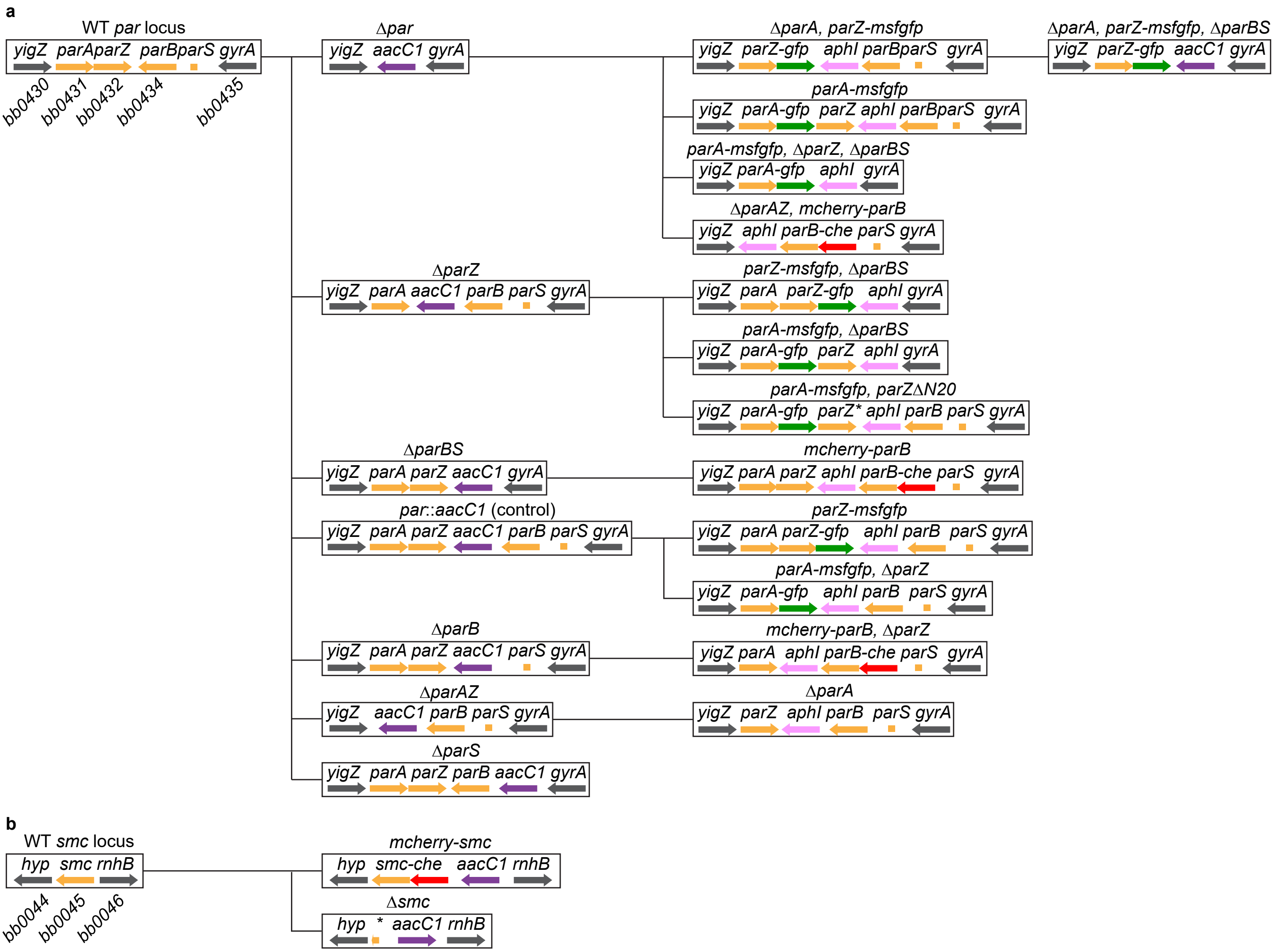

## SUPPLEMENTARY REFERENCES

1 Rodionov, O., Lobocka, M. & Yarmolinsky, M. Silencing of genes flanking the P1 plasmid centromere. *Science* **283**, 546-549 (1999).

2 Dabrazhynetskaya, A., Sergueev, K. & Austin, S. Species and incompatibility determination within the P1*par* family of plasmid partition elements. *J. Bacteriol.* **187**, 5977-5983 (2005).

3 Livny, J., Yamaichi, Y. & Waldor, M. K. Distribution of centromere-like *parS* sites in bacteria: insights from comparative genomics. *J. Bacteriol.* **189**, 8693-8703 (2007).

4 Li, Y. & Austin, S. The P1 plasmid is segregated to daughter cells by a 'capture and ejection' mechanism coordinated with *Escherichia coli* cell division. *Mol. Microbiol.* **46**, 63-74 (2002).

5 Takacs, C. N., Kloos, Z. A., Scott, M., Rosa, P. A. & Jacobs-Wagner, C. Fluorescent proteins, promoters, and selectable markers for applications in the Lyme disease spirochete *Borrelia burgdorferi*. *Appl. Environ. Microbiol.* **84**, e01824-18 (2018).

6 Rego, R. O., Bestor, A. & Rosa, P. A. Defining the plasmid-borne restriction-modification systems of the Lyme disease spirochete *Borrelia burgdorferi*. *J. Bacteriol.* **193**, 1161-1171 (2011).

7 Hayes, B. M. *et al.* Regulatory protein BBD18 of the lyme disease spirochete: essential role during tick acquisition? *mBio* **5**, e01017-14 (2014).

8 Purser, J. E. & Norris, S. J. Correlation between plasmid content and infectivity in *Borrelia burgdorferi*. *Proc. Natl. Acad. Sci. USA.* **97**, 13865-13870 (2000).

9 Jacobs, M. B., Norris, S. J., Phillippi-Falkenstein, K. M. & Philipp, M. T. Infectivity of the highly transformable BBE02- lp56- mutant of *Borrelia burgdorferi*, the Lyme disease spirochete, via ticks. *Infect. Immun.* **74**, 3678-3681 (2006).

10 Kawabata, H., Norris, S. J. & Watanabe, H. BBE02 disruption mutants of *Borrelia burgdorferi* B31 have a highly transformable, infectious phenotype. *Infect. Immun.* **72**, 7147-7154 (2004).

11 Jewett, M. W. *et al.* Genetic basis for retention of a critical virulence plasmid of *Borrelia burgdorferi*. *Mol. Microbiol.* **66**, 975-990 (2007).

12 Sadziene, A., Wilske, B., Ferdows, M. S. & Barbour, A. G. The cryptic *ospC* gene of *Borrelia burgdorferi* B31 is located on a circular plasmid. *Infect. Immun.* **61**, 2192-2195 (1993).

13 Casjens, S., van Vugt, R., Tilly, K., Rosa, P. A. & Stevenson, B. Homology throughout the multiple 32-kilobase circular plasmids present in Lyme disease spirochetes. *J. Bacteriol.* **179**, 217-227 (1997).

14 Iyer, R. *et al.* Stage-specific global alterations in the transcriptomes of Lyme disease spirochetes during tick feeding and following mammalian host adaptation. *Mol. Microbiol.* **95**, 509-538 (2015).

15 Dunham-Ems, S. M. *et al.* Live imaging reveals a biphasic mode of dissemination of *Borrelia burgdorferi* within ticks. *J. Clin. Invest.* **119**, 3652-3665 (2009).

16 Fazzino, L., Tilly, K., Dulebohn, D. P. & Rosa, P. A. Long-term survival of *Borrelia burgdorferi* lacking the hibernation promotion factor homolog in the unfed tick vector. *Infect. Immun.* **83**, 4800-4810 (2015).

17 Tilly, K., Bestor, A. & Rosa, P. A. Functional equivalence of OspA and OspB, but not OspC, in tick colonization by *Borrelia burgdorferi*. *Infect. Immun.* **84**, 1565-1573 (2016).

18 Chu, C. Y. *et al.* Function of the *Borrelia burgdorferi* FtsH homolog is essential for viability both in vitro and in vivo and independent of HflK/C. *mBio* **7**, e00404-00416 (2016).
19 Di, L. *et al.* BorreliaBase: a phylogeny-centered browser of *Borrelia* genomes. *BMC Bioinformatics* **15**, 233 (2014).
20 Burgdorfer, W. *et al.* Lyme disease-a tick-borne spirochetosis? *Science* **216**, 1317-1319 (1982).
21 Fraser, C. M. *et al.* Genomic sequence of a Lyme disease spirochaete, *Borrelia burgdorferi*. *Nature* **390**, 580-586 (1997).
22 Casjens, S. *et al.* A bacterial genome in flux: the twelve linear and nine circular extrachromosomal DNAs in an infectious isolate of the Lyme disease spirochete *Borrelia burgdorferi*. *Mol. Microbiol.* **35**, 490-516 (2000).
23 Steere, A. C. *et al.* The spirochetal etiology of Lyme disease. *N. Engl. J. Med.* **308**, 733-740 (1983).
24 Schutzer, S. E. *et al.* Whole-genome sequences of thirteen isolates of *Borrelia burgdorferi*. *J. Bacteriol.* **193**, 1018-1020 (2011).
25 Casjens, S. R. *et al.* Genome stability of Lyme disease spirochetes: comparative genomics of *Borrelia burgdorferi* plasmids. *PLoS One* **7**, e33280 (2012).
26 Piesman, J., Mather, T. N., Sinsky, R. J. & Spielman, A. Duration of tick attachment and *Borrelia burgdorferi* transmission. *J. Clin. Microbiol.* **25**, 557-558 (1987).
27 Schwan, T. G., Burgdorfer, W., Schrumpf, M. E. & Karstens, R. H. The urinary bladder, a consistent source of *Borrelia burgdorferi* in experimentally infected white-footed mice (*Peromyscus leucopus*). *J. Clin. Microbiol.* **26**, 893-895 (1988).
28 Lin, T. *et al.* Analysis of an ordered, comprehensive STM mutant library in infectious *Borrelia burgdorferi*: insights into the genes required for mouse infectivity. *PLoS One* **7**, e47532 (2012).
29 Chan, K., Alter, L., Barthold, S. W. & Parveen, N. Disruption of *bbe02* by insertion of a luciferase gene increases transformation efficiency of *Borrelia burgdorferi* and allows live imaging in Lyme disease susceptible C3H mice. *PLoS One* **10**, e0129532 (2015).
30 Chaconas, G., Stewart, P. E., Tilly, K., Bono, J. L. & Rosa, P. Telomere resolution in the Lyme disease spirochete. *EMBO J.* **20**, 3229-3237 (2001).
31 Bestor, A. *et al.* Use of the Cre-*lox* recombination system to investigate the lp54 gene requirement in the infectious cycle of *Borrelia burgdorferi*. *Infect. Immun.* **78**, 2397-2407 (2010).
32 Stewart, P. E., Thalken, R., Bono, J. L. & Rosa, P. Isolation of a circular plasmid region sufficient for autonomous replication and transformation of infectious *Borrelia burgdorferi*. *Mol. Microbiol.* **39**, 714-721 (2001).
33 Grimm, D. *et al.* Experimental assessment of the roles of linear plasmids lp25 and lp28-1 of *Borrelia burgdorferi* throughout the infectious cycle. *Infect. Immun.* **72**, 5938-5946 (2004).
34 Stewart, P. E. & Rosa, P. A. Transposon mutagenesis of the Lyme disease agent *Borrelia burgdorferi*. *Methods Mol. Biol.* **431**, 85-95 (2008).
35 Blevins, J. S., Revel, A. T., Smith, A. H., Bachlani, G. N. & Norgard, M. V. Adaptation of a luciferase gene reporter and *lac* expression system to *Borrelia burgdorferi*. *Appl. Environ. Microbiol.* **73**, 1501-1513 (2007).
