# Supplementary material for: Polyploidy, regular patterning of genome copies, and unusual control of DNA partitioning in the Lyme disease spirochete
Source: Nat Commun. 2022 Nov 22;13:7173. doi: 10.1038/s41467-022-34876-4 (PMC9712426; doi:10.1038/s41467-022-34876-4)
Supplement: Supplementary file 3 — Description of Additional Supplementary Files [file 41467_2022_34876_MOESM3_ESM.pdf]

Description of Additional Supplementary Files:

Supplementary Dataset 1: Supplementary Data 1 contains information regarding the B. burgdorferi strains used in this study (Worksheet 1), the plasmids used in this study (Worksheet 2) and sequences of oligonucleotide primers used for plasmid generation (Worksheet 3). Worksheet-specific legends for each applicable column are provided in the first row below the column title.

Supplementary Dataset 2: Supplementary Data 2 contains the following information regarding the imaging experiments analyzed in this study. Worksheet 1 lists all the imaging experiments, including information on culture density, number of cells analyzed, and analysis parameters used for spot detection. Worksheet 2 lists experimental details about the cultures used for ChIP-seq experiments and WGS as well as the GEO access codes for each sample. Worksheet 3 summarized the number of replicates and the number of cells analysed in order to generate the indicated figure panels
