# Supplementary material for: Polyploidy, regular patterning of genome copies, and unusual control of DNA partitioning in the Lyme disease spirochete
Source: Nat Commun. 2022 Nov 22;13:7173. doi: 10.1038/s41467-022-34876-4 (PMC9712426; doi:10.1038/s41467-022-34876-4)
Supplement: Supplementary file 6 — Reporting Summary [file 41467_2022_34876_MOESM6_ESM.pdf]

nature research

Corresponding author(s): Xindan Wang; Christine Jacobs-Wagner

Last updated by author(s): 11/05/2022

# Reporting Summary

Nature Research wishes to improve the reproducibility of the work that we publish. This form provides structure for consistency and transparency in reporting. For further information on Nature Research policies, see our Editorial Policies and the Editorial Policy Checklist.

## Statistics

For all statistical analyses, confirm that the following items are present in the figure legend, table legend, main text, or Methods section.

| n/a | Confirmed | |
|---|---|---|
| ☐ | ☒ | The exact sample size (*n*) for each experimental group/condition, given as a discrete number and unit of measurement |
| ☐ | ☒ | A statement on whether measurements were taken from distinct samples or whether the same sample was measured repeatedly |
| ☒ | ☐ | The statistical test(s) used AND whether they are one- or two-sided<br>*Only common tests should be described solely by name; describe more complex techniques in the Methods section.* |
| ☒ | ☐ | A description of all covariates tested |
| ☒ | ☐ | A description of any assumptions or corrections, such as tests of normality and adjustment for multiple comparisons |
| ☐ | ☒ | A full description of the statistical parameters including central tendency (e.g. means) or other basic estimates (e.g. regression coefficient) AND variation (e.g. standard deviation) or associated estimates of uncertainty (e.g. confidence intervals) |
| ☒ | ☐ | For null hypothesis testing, the test statistic (e.g. *F*, *t*, *r*) with confidence intervals, effect sizes, degrees of freedom and *P* value noted<br>*Give P values as exact values whenever suitable.* |
| ☒ | ☐ | For Bayesian analysis, information on the choice of priors and Markov chain Monte Carlo settings |
| ☒ | ☐ | For hierarchical and complex designs, identification of the appropriate level for tests and full reporting of outcomes |
| ☒ | ☐ | Estimates of effect sizes (e.g. Cohen's *d*, Pearson's *r*), indicating how they were calculated |

*Our web collection on statistics for biologists contains articles on many of the points above.*

## Software and code

Policy information about availability of computer code

| | |
|---|---|
| Data collection | Microscopy data was collected using NIS Elements version 4.20 |
| Data analysis | Microscopy data was analyzed using Oufti and FiJi software, which are published software packages and are referenced in the text appropriately. Custom MATLAB and R code was deposited at GitHub as described in the manuscript. The Github links are: https://https://github.com/JacobsWagnerLab/published and https://github.com/xindanwanglab/takacs-2022-natcomm. DOI links are provided in the mansucript. MATLAB R2019a, Graphpad Prism 9.3.1, and GeneiousR10.0.0 software were also used. |

For manuscripts utilizing custom algorithms or software that are central to the research but not yet described in published literature, software must be made available to editors and reviewers. We strongly encourage code deposition in a community repository (e.g. GitHub). See the Nature Research guidelines for submitting code & software for further information.

## Data

Policy information about availability of data

All manuscripts must include a data availability statement. This statement should provide the following information, where applicable:

- Accession codes, unique identifiers, or web links for publicly available datasets
- A list of figures that have associated raw data
- A description of any restrictions on data availability

The reference B. burgdorferi B31 genome is available from NCBI (GenBank assembly accession code GCA_000008685.2). The sequence of the codon-optimized parBP1 gene was deposited with Genbank (accession code ON321895, release date Nov 8, 2022). The ChIP-Seq and WGS data generated in this study are deposited in the NCBI Gene Expression Omnibus platform and are publicly available through GEO Series accession number GSE202255 (https://www.ncbi.nlm.nih.gov/geo/query/acc.cgi?acc=GSE202255). The accession numbers for each sample can be found in Supplementary Data 2 Worksheet 2. Source data are provided with this paper. All other reasonable requests for raw data should be directed by email to the corresponding authors.

# Field-specific reporting

Please select the one below that is the best fit for your research. If you are not sure, read the appropriate sections before making your selection.

☒ Life sciences ☐ Behavioural & social sciences ☐ Ecological, evolutionary & environmental sciences

For a reference copy of the document with all sections, see nature.com/documents/nr-reporting-summary-flat.pdf

# Life sciences study design

All studies must disclose on these points even when the disclosure is negative.

| | |
|---|---|
| Sample size | Microscopy images were randomly collected to obtain sufficient number of cells for each replicate. The number of cells analyzed for each condition is provided in Supplementary Data 2 |
| Data exclusions | Data was excluded based on neutral principles. For example, cells in which spot detection failed due to the cells being out of focus were removed, as described in the manuscript |
| Replication | All replication attempts were successful. The detailed information about the number of replication and number of cells or samples used is available in Supplementary Data 2, Worksheets 1 and 3, which is noted in the relevant figures. |
| Randomization | Microscopy images were randomly collected in the following manner. For each strain and for each replicate, the cells were spotted on an agarose pad, a region of the pad with good cell density was identified, and then images were acquired automatically along a predefined grid of positions. Image acquisition was done without prior knowledge of the signal within the imaged cells, and was therefore random.<br><br>There was no random allocation into experimental groups. In some cases, each strain was its own experimental group (e.g., comparing WT with parZ deletion strain). In other cases, a given treatment condition (e.g., piperacillin, 24 h) was its own experimental group. Lack of a purposeful allocation into experimental groups and the types of questions we asked and analyses we performed in this study do not require control of any covariates.<br><br>Otherwise, the experimental samples were grouped based on the strain used or treatment condition used. See for example the ChIP-seq and growth curve experiments. |
| Blinding | Here we are studying bacteria cells. The investigators were aware of the genotypes of the bacteria during data acquisition and analyses. |

# Reporting for specific materials, systems and methods

We require information from authors about some types of materials, experimental systems and methods used in many studies. Here, indicate whether each material, system or method listed is relevant to your study. If you are not sure if a list item applies to your research, read the appropriate section before selecting a response.

## Materials & experimental systems

| n/a | Involved in the study | |
|---|---|---|
| ☐ | ☒ | Antibodies |
| ☒ | ☐ | Eukaryotic cell lines |
| ☒ | ☐ | Palaeontology and archaeology |
| ☐ | ☒ | Animals and other organisms |
| ☒ | ☐ | Human research participants |
| ☒ | ☐ | Clinical data |
| ☒ | ☐ | Dual use research of concern |

## Methods

| n/a | Involved in the study | |
|---|---|---|
| ☐ | ☒ | ChIP-seq |
| ☒ | ☐ | Flow cytometry |
| ☒ | ☐ | MRI-based neuroimaging |

## Antibodies

| | |
|---|---|
| Antibodies used | anti-GFP (PMID: 10611287) and anti-mCherry (PMID: 26253537) antibodies were used for ChIP-seq studies. All of these antibodies were requested from previously published sources as cited. |
| Validation | We did not re-validate these published, non-commercial antibodies. We cited the original publications in which their generation was described. |

## Animals and other organisms

Policy information about studies involving animals; ARRIVE guidelines recommended for reporting animal research

| | |
|---|---|
| Laboratory animals | Use of lab animals (mice and ticks) is described in the methods section. Four to eight weeks-old female RML mice, an out-bred strain |

| | |
|---|---|
| Laboratory animals | of Swiss-Webster Mus musculus lab mice were used and housed at 72F+/-3F, 50%+/- 10% humidity and a 12-h ON / 12-h OFF light/dark cycle. Larval Ixodes scapularis ticks were purchased from Oklahoma State University. |
| Wild animals | No wild animals were used in the study. |
| Field-collected samples | No field-collected samples were used in the study. |
| Ethics oversight | Study was approved by the Rocky Mountain Laboratories, NIAID, NIH IACUC. The facility has AAALAC accreditation. |

Note that full information on the approval of the study protocol must also be provided in the manuscript.

# ChIP-seq

## Data deposition

☒ Confirm that both raw and final processed data have been deposited in a public database such as GEO.

☐ Confirm that you have deposited or provided access to graph files (e.g. BED files) for the called peaks.

| | |
|---|---|
| Data access links<br>*May remain private before publication.* | https://www.ncbi.nlm.nih.gov/geo/query/acc.cgi?acc=GSE202255 |
| Files in database submission | Raw and processed data are submitted to the database. The list of individual samples can be found in Supplementary Data 2. We did not use software for peak calling. Therefore, BED files were not generated and the requirement for depositing BED files is not applicable. |
| Genome browser session<br>(e.g. UCSC) | https://www.dropbox.com/sh/n2txd34caiog7i5/AAAQKuC1NekzNx9aGukRehi1a?dl=0 |

## Methodology

| | |
|---|---|
| Replicates | Two biological experiments were done for each ChIP-seq and WGS, except for strain CJW_Bb524, where one replicate was analyzed. Independent ChIP-seq and imaging approaches yielded the same conclusion. |
| Sequencing depth | ChIP-seq reads were sequenced using paired-end sequencing with 42nt per read. Over two million raw reads and uniquely mapped reads were generated for each sample. |
| Antibodies | anti-GFP and anti-mCherry antibodies were used for ChIP-seq. They were requested from previously published sources as cited in the manuscript. |
| Peak calling parameters | Not applicable. Genome-wide distribution of reads were plotted. Peak calling was not performed. |
| Data quality | This study did not involve peak calling. Genome-wide distribution of reads were plotted. We made sure that every sample had greater than two million reads. |
| Software | Reads were mapped to the genome using CLC genomics workbench (Qiagen). The data were plotted using R. |
